# Next-generation sequencing analysis of multiplex families with atypical psychosis

**DOI:** 10.1038/s41398-018-0272-x

**Published:** 2018-10-15

**Authors:** Tatsushi Okayama, Yasuyuki Hashiguchi, Hiroki Kikuyama, Hiroshi Yoneda, Tetsufumi Kanazawa

**Affiliations:** 1 Department of Neuropsychiatry, Osaka Medical College, Osaka, Japan; 2 Department of Biology, Osaka Medical College, Osaka, Japan; 3 Shin-Abuyama Hospital, Osaka, Japan; 4 Department of Psychiatry, The University of Melbourne, Melbourne, VIC Australia; 5 The Florey Institute of Neuroscience and Mental Health, Melbourne, VIC Australia

## Abstract

Atypical psychosis (similar to acute and transient psychotic disorder, brief psychotic disorder) is highly heritable, but the causal genes remain unidentified. We conducted whole-genome sequencing on multiplex Japanese families with atypical psychosis. The patient group of interest shows acute psychotic features including hallucinations, delusions, and catatonic symptoms while they often show good prognosis after the onset. In addition to the next-generation analysis, HLA typing has been conveyed to check the similarity with autoimmune disease, such as systemic lupus erythematosus (SLE). Shared causal polymorphisms in the Deleted in Colorectal Carcinoma, Netrin 1 receptor (DCC) gene were found in one multiplex family with three patients, and variants in the RNA 3′-Terminal Phosphate Cyclase (RTCA) and One Cut Homeobox 2 (ONECUT2) genes were found to be shared in seven patients. Next-generation sequencing analysis of the MHC region (previously suggested to be a hot region in atypical psychosis) using HLA typing (HLA-DRB1) revealed a common vulnerability with SLE (systemic lupus erythematosus) among five patients. This finding demonstrates the shared etiology between psychotic symptoms and autoimmune diseases at the genetic level. Focusing on a specific clinical phenotype is key for elucidating the genetic factors that underlie the complex traits of psychosis.

## Introduction

Atypical psychosis is a taxonomic name that has been used by Japanese psychiatrists since Dr. Mitsuda advocated its use^1^. Atypical psychosis patients are classified as having brief psychotic disorders (298.8)^2^ or acute and transient psychotic disorders (F23)^3^ in the current nosological system. As experienced by those with these disorders, patients with atypical psychoses experience acute onset and hallucinations and/or mood disturbances during the acme phase. Patients with this disorder worsen periodically but can live normal lives after these brief exacerbations and may not remember the experience. Most of these patients are middle-aged females and do not require continuous drug treatment after onset, although some patients are vulnerable to relapse. Patients with atypical psychosis seldom show cognitive decline in the course of the disease, while patients with schizophrenia (SZ) and bipolar disorder (BD) often exhibit cognitive decline. Moreover, the estimated heritability in atypical psychosis and similar disorders is slightly higher than that reported for SZ and BD^4,5^. Our group focused on a specific patient group with atypical psychosis. Similar to brief psychotic episodes, “atypical psychosis” has been defined elsewhere (Supplementary Table 1)^6^. In addition, a genome-wide association study (GWAS) analysis was conducted in 47 patients with this disorder. Our main finding was that the causal variants for these atypical psychoses are located primarily within the MHC (major histocompatibility complex) region, and the genetic information in these patients is more similar to the genetic information reported for SZ than the genetic information reported for BD^6^. However, no single SNP achieved genome-wide significance (*p* < 5 × 10^−8^). In the current report, next-generation sequencing (NGS) was applied to a set of multiplex families with atypical psychosis. NGS has the advantage of sequencing the entire genome in depth^7^. Thus, distinguishing the phenotype enables the causal gene(s) to be detected (e.g., the *SH3TC2* gene for Charcot–Marie–Tooth Neuropathy)^8^. Our primary aim was to reveal the gene(s) responsible for atypical psychosis via NGS, which enables rare mutations to be detected, including single-nucleotide and insertion/deletion polymorphisms, even in the MHC region. The genetic findings in patients with this rare phenotype will shed light on the current psychiatric typology.

## Methods

### Study participants

**Fig. 1:**
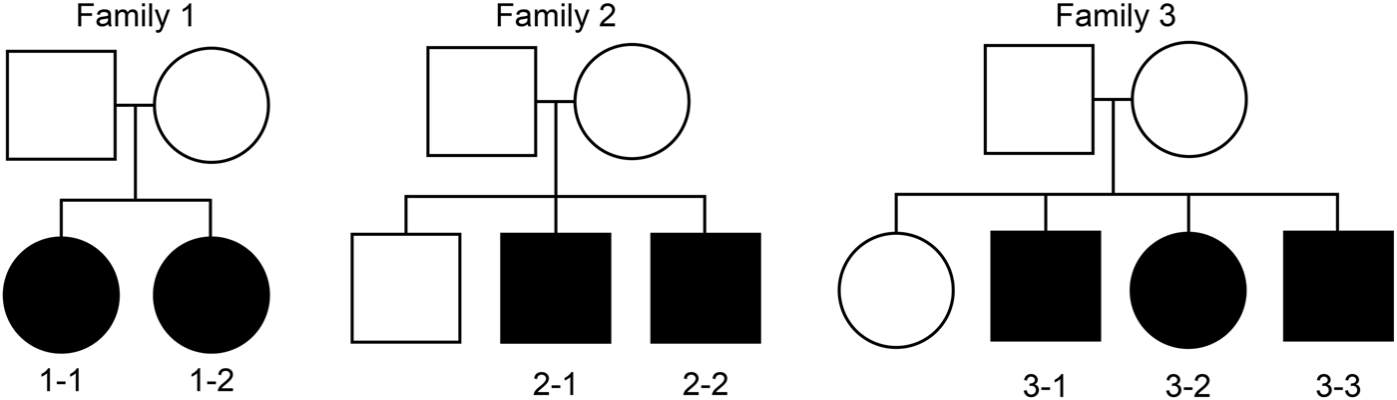
The demographic scheme of multiplex families

Seven affected individuals from three multiplex families were analyzed (Fig. 1 and Supplementary Table 2). None of the parents in the analyzed families were affected by the psychiatric disorder. Subjects were excluded if they had also been diagnosed with an intellectual disability. All subjects live in Japan and self-identify as Japanese. Written informed consent was obtained from all participants, and this study was approved by the institutional review board at Osaka Medical College. Based on the consensus of at least two experienced psychiatrists, the seven affected patients were diagnosed using the criteria for atypical psychosis (Supplementary Table 1**)**^6^. The patients showed no abnormal results, such as inflammatory symptoms or abnormal electroencephalogram results, upon physical examination. None of the analyzed patients had a history of autoimmune disease or showed symptoms of such disease.

### Genotyping procedures

#### Whole-genome sequencing and sequence alignment

Whole-genome sequencing was performed using HiSeq 2500 (Illumina, San Diego, CA) according to the manufacturer’s protocol, with a target coverage of 30× (100 bp paired-end reads). The genome sequences for each individual were assembled using the following procedure. First, the HiSeq sequence read quality was assessed, and ambiguous (i.e., low quality) reads and adaptor sequences were removed using the script FaQCs.pl^9^. Second, the sequencing reads were mapped to the Japanese reference genome (hg38 + decoy JRGv1) provided by the Tohoku Medical Megabank Organization (ToMMo)^10^ using Barrows-Wheeler Aligner (BWA) version 0.7.2-r1039^11^, and the resulting SAM files were converted to BAM files and sorted using SAM tools version 0.1.20^12^ for further analysis. Third, to map sequences, mate-pair information was verified, and potential duplicate PCR reads were excluded using PICARD-Tools version 2.2.1 (http://broadinstitute.github.io/picard/). Genome Analysis Toolkit (GATK) version 3.5^13^ was used to perform local realignments and to map quality score recalibration to produce cleaned BAM files.

#### Variant detection, quality controls, and annotation

Variants, including single-nucleotide variants (SNVs) and short insertions and deletions (INDELs), were determined using GATK. The 1000 Genomes database (ver. August 2015)^14^ was used as a reference panel, and 7,836,147 polymorphisms were detected. Low-quality variants were filtered and excluded using the programs implemented in GATK. Individual variants were annotated using the ANNOVAR program package^15^.

#### Detection of nucleotide variants linked to atypical psychosis

To identify the candidate variants of atypical psychosis, we narrowed the variants by applying the following three criteria: (1) the variants were shared among two or three affected siblings within a family (i.e., families 1–3, see Fig. 1) but not with other families; (2) the variants were shared among affected siblings within two families (i.e., families 1 and 2, families 1 and 3, and families 2 and 3) but not with the remaining family; or (3) the variants were shared with all seven affected individuals analyzed in this study. For all criteria, we first selected the rare variants in which alternative allele frequencies were less than 0.01 in the reference panel (1000-genome sequences from all ethnicity) since the assumed prevalence of the disorder of interest is ~0.002 or less^16^. In this study, microRNA and long intergenic non-protein-coding RNA genes were excluded from the analysis. In whole-genome re-sequencing by short reads, length variations occur frequently in homopolymers (=stretches of the same nucleotide) and short tandem repeats (STRs) caused by experimental and/or computational errors^17^. The criterion 3 described above cannot exclude this type of errors from the selected rare variants because these errors are frequently shared among all seven individuals. To validate the rare insertion/deletion variants shared among the seven individuals, the nucleotide sequences around the variants were examined manually using the Integrative Genome Viewer (IGV) version 2.3. Variants were excluded from the analysis if they were a part of homopolymer runs or STRs. In addition, to confirm whether the variants were located within segmentally duplicated genomic regions, BLASTN searches against the human genome (hg38) were conducted using 5′ and 3′ nucleotide sequences around each variant (102–195 bp) as queries. If the query sequences hit two or more genomic regions with >90% nucleotide identity, the variants within the query were considered to be artifacts caused by the mapping of slightly different duplicated sequences in one region and were thus excluded from the analysis. In this study, we only considered homozygous variants because no parents in the three families have shown similar psychiatric phenotypes. The variants were selected and narrowed using the R version 3.2.1 software package (https://www.r-project.org/).

#### Super high-resolution typing in the MHC region

High-resolution typing was performed on MHC expanded regions in all analyzed samples since previous GWAS work on forty-seven individuals revealed this region’s possible involvement. In addition, general reading by NGS is limited because of its complex structure. HLA (HLA-A, B, C, DRB1, DRB3/4/5, DQA1, DQB1, DPA1, and DPB1) four-digit allele typing was performed using the ScisGo HLA (Scisco Genetics, Inc., WA) method.

## Results

### Variants within one family and shared with two of three families

**Fig. 2:**
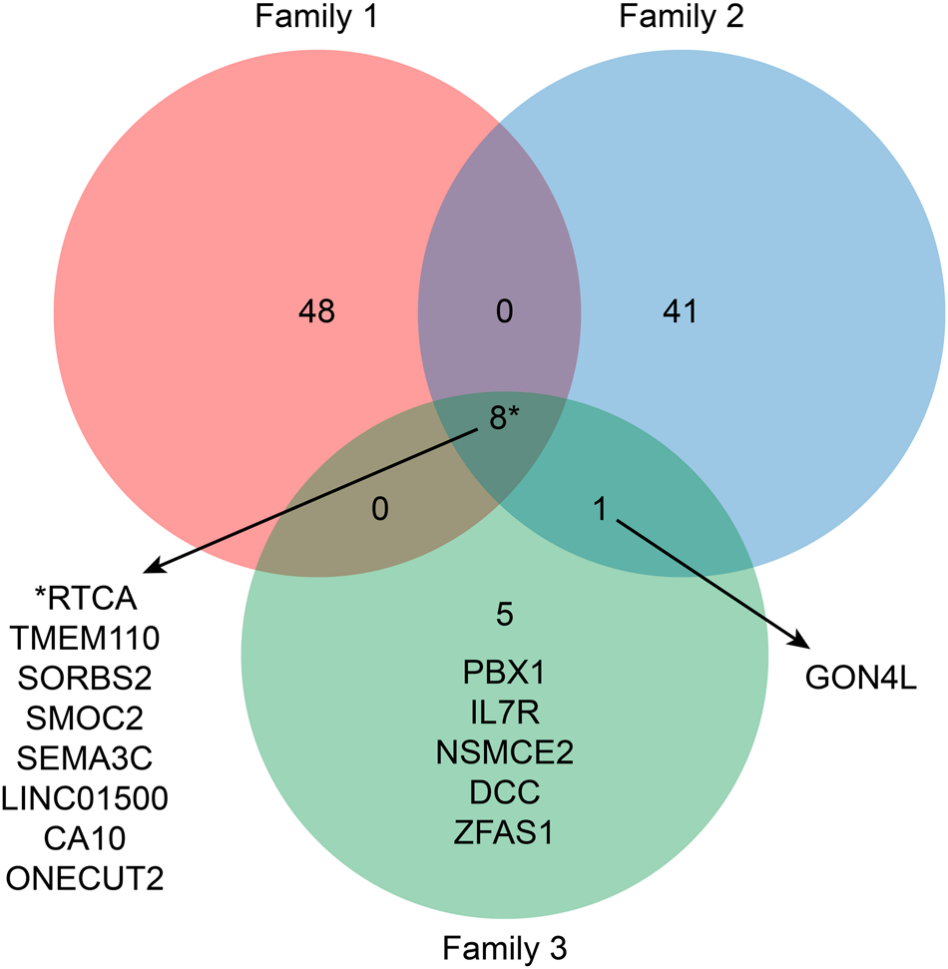
Venn diagram

First, common variants in the two siblings in two families (Case numbers 1–1, 1–2 in family 1 and 2–1, 2–2 in family 2) were analyzed. In family 1, 96 variants in 48 genes were identified (Fig. 2, Supplementary Tables 3 and 4) and in family 2, 93 variants in 41 genes were detected (Fig. 2, Supplementary Tables 5 and 6).

Second, common variants in three siblings (Case numbers 3–1, 3–2, and 3–3 in family 3 were analyzed. Thirteen loci were detected as causative variants (11 SNVs and 2 INDELs; detailed description is in Supplementary Table 7). Eight loci out of them were located within intronic or 3′-UTR regions of the genes shown in Table 1 and Fig. 2, no functional (non-synonymous) variants being found in this analysis. Remaining five variants were located in intergenic genomic regions (see Supplementary Table 7).

**Table 1:** Genes with detected variants across three affected individuals within family 3 (excluding microRNA and long intergenic non-protein-coding RNA)

Gene	Cytogenic location	Polymorphisms	Ref	Alt	Function	Gene function
PBX1	1q23.3	rs117586882	A	G	Intronic	Encodes a nuclear protein in the PBX homeobox family
IL-7R	5p13.2	rs76614394	C	T	3′-UTR	A receptor for interleukin 7, cause of severe combined immunodeficiency
NSMCE2	8q24.13	rs200273856	ACT	–	Intronic	Small ubiquitin-related modifier, nuclear transport, transcription, and DNA repair
DCC	18q21.2	rs142009962	C	T	Intronic	Netrin-1 receptor, axon guidance of neural cells
DCC	18q21.2	rs143136177	G	A	Intronic	
DCC	18q21.2	rs149368621	C	T	Intronic	
DCC	18q21.2	rs148469099	G	A	Intronic	
ZFAS1	20q13.13	rs199835143	CTC	—	ncRNA_intronic	Unknown

Third, we tried to identify the common variants between two out of the 3 families used in this study. No common variants were found between the patients in families 1 and 2 and families 1 and 3, respectively (Fig. 2). One common variant was detected between patients in families 2 and 3 (Fig. 2). This variant was a 2 bp INDEL in the intronic region of GON4L (GON4-like protein) gene.

### Variants shared by all seven individuals

More than 600,000 variants were found to be shared in the DNA information of these affected seven individuals. From these variants, using the same method described above, we selected 82 variants (16 SNVs and 66 INDELs; Supplementary Table 8). After removal of possible artifacts (i.e., variants located in STRs, homopolymer runs, and/or duplicated genomic regions) and variants without specific RefSNP (rs) numbers, 18 variants (4 SNVs and 14 INDELs) were remained (Table 2). Eight of these variants were located within protein-coding or ncRNA genes (Fig. 2), including an INDEL in the 5′-UTR region of the *RTCA* (RNA 3′-terminal phosphate cyclase) gene (rs57195277) and a SNP in the 3′-UTR region of the *ONECUT2* (one cut homeobox 2) gene (rs143974794). Detailed data of 82 genes are listed in Supplementary Table 8.

**Table 2:** Selected shared variants within affected seven patients (variants within protein-coding genes)

Gene	Cytogenic location	Polymorphisms	Start	End	Ref	Alt	Function	1000genome_all_ethnicity	1000genome_east_Asia
ADGRL2;LINC01362	1p31.1	rs61765064	82331076	82331076	A	G	intergenic	0.0061901	0.0149
RTCA	1p21.2	rs57195277	1E + 08	1E + 08	—	A	UTR5	0.000798722	0.001
TMEM110;TMEM110-MUSTN1	3p21.1	rs397703917	52843631	52843631	—	A	intronic	0.00559105	NA
AADAT;LINC01612	4q33	rs11268329	1.7E + 08	1.7E + 08	—	CTTCTCTTGGC	intergenic	0.00119808	NA
SORBS2	4q35.1	rs1499016	1.86E + 08	1.86E + 08	G	T	intronic	0.00858626	0.001
SMOC2	6q27	rs140322343	1.69E + 08	1.69E + 08	—	CTCCTTCCAAGGCCTCGCCCTGAGTGGCCGA	intronic	0.00279553	NA
SEMA3C	7q21.11	rs141461553	80747025	80747025	—	AT	intronic	0.00339457	0.0119
ADRA2A;GPAM	10q25.2	rs113565291	1.12E + 08	1.12E + 08	—	TTTAA	intergenic	0.00139776	NA
FOXI2;CLRN3	10q26.2	rs386372808	1.28E + 08	1.28E + 08	—	T	intergenic	0.00519169	0.0149
TMEM135;LOC105369423	11q14.2	rs71043634	87707788	87707788	—	T	intergenic	0.00838658	0.0268
ANKRD10;LINC00431	13q34	rs10693206	1.11E + 08	1.11E + 08	—	AACTTT	intergenic	0.00179712	0.003
NID2;PTGDR	14q22.1	rs3032506	52244314	52244318	TACTT	—	intergenic	0.00239617	NA
LINC01500	14q23.1	rs71107991	58859573	58859573	—	A	ncRNA_intronic	0.00878594	0.0278
SPATA8;LINC02254	15q26.2	rs56058536	97206586	97206586	C	G	intergenic	0.00259585	0.006
SMPD3;ZFP90	16q22.1	rs75455089	68463627	68463627	—	AAAGTGCCTACCC	intergenic	0.00119808	0.003
CA10	17q21.33	rs202130965	51734041	51734041	—	TCAA	intronic	0.00319489	0.002
ONECUT2	18q21.31	rs143974794	57477268	57477268	—	ATA	UTR3	0.00299521	0.003
MIR3687–1;TEKT4P2	21p11.2	rs555385637	9061763	9061763	T	G	intergenic	0.000399361	NA

### HLA-DRB1

Detailed data for all patients are shown in Supplementary Table 9, and HLA-DRB1 is shown in Table 3. A certain HLA type on DRB1 was revealed to be associated with systemic lupus erythematosus (SLE) onset in the Japanese population, and the vulnerable HLA types (08:02, 09:01, and 15:01 on HLA-DRB1) match. Significant correlations were found between vulnerable HLA types in SLE in these patients.

**Table 3:** HLA-DRB1 data on High–Resolution Typing

	HLA-DRB1	
Family 1–1	04:10:01	11:01:01
Family 1–2	11:01:01	15:01:01
Family 2–1	08:03:02	09:01:02
Family 2–2	04:05:01	15:01:01
Family 3–1	08:02:01	09:01:02
Family 3–2	08:02:01	09:01:02
Family 3–3	09:01:02	15:02:01

## Discussion

The heritability of mental disorders, such as SZ and BD, is estimated to be ~0.8–0.85^18^, and the search for disease susceptibility genes is ongoing worldwide. In the last decade, dozens of genome-wide association studies (GWAS) have been conducted to identify causal genes for psychiatric disorders. More than one hundred susceptible loci have been associated with SZ^19,20^, although the estimated maximum relative risk of a single SNP is only 1.2^21^. This era has generated significant scientific breakthroughs, including the following findings: (1) no single genetic variant (SNP/CNV) explains the entire etiology of SZ or BD; (2) these two disorders exhibit high genetic overlap; and (3) broadly defined phenotypes (symptoms or clinical courses) require larger sample sizes for causal gene detection, although no evidence exists for finding such a gene in the future.

To our knowledge, this is the first report describing the genetic analysis of atypical psychosis (similar to acute and transient psychosis, brief psychosis) by NGS. This disorder is rarely encountered in practical psychiatry (~10% or less of inpatients in psychiatric wards), and we believe a rare variant mutation is needed to clarify the etiology of this disorder. To reduce genetic noise, affected patients in multiplex families were analyzed in the current design.

The common clinical features of participants were the sudden onset (less than 2 weeks) of psychotic status, including emotional turmoil, memory confusion, and/or hallucinations or delusions. With antipsychotic drugs, the patients recovered safely to their pre-onset status. Most patients exhibited relapse after the first admission with the same psychotic features, although they recovered safely.

Historically, patients with sudden onset psychotic symptoms have received much attention, although no clear definition has been established due to low longitudinal diagnostic stability^22^. For example, the following are nosologically synonymous with “Brief Psychotic Disorder (DSM-5)”: “Acute and transient psychotic disorder (ICD-10)” and “Bouffée délirante” with “Atypical Psychosis” as it is defined in Japan. According to the epidemiological survey, a higher incidence was reported within the family members who had affected patients^23,24^, thus providing a rationale for identifying the causal gene.

In one multiplex family (family 3; Fig. 1), we found that 8 loci in 5 genes were shared among 3 affected individuals but not normal controls (Fig. 2; Supplementary Table 7). The deleted in colorectal carcinoma (*DCC*) gene, which contains four SNPs, encodes the netrin-1 receptor. This encoded protein guides axons in brain development, and altering the DCC protein level through coexpression with microRNA leads to mood symptoms in animal models^25^. Recently, biallelic loss-of-function mutations in this gene were found to cause developmental split-brain syndrome^26^. Recent large-scale GWAS analysis focusing on the mood instability reported the variance on *DCC* gene as an associated loci^27^.

Pre-B-cell leukemia homeobox 1 (PBX1) and interleukin 7 receptor (IL-7R) are relevant to the immune system, specifically IL-7, which is a main cytokine. IL-7R knockout mice exhibit immunodeficiency^28^, and the robust relationship between IL-7R and psychotic disorders remains to be investigated, as with MHC region involvement.

In addition to family 3, families 1 and 2 were investigated. Since only two affected members were genotyped, 96 variants in 48 genes for Family 1 and 93 variants in 41 genes for Family 2 were detected as shared genes (Supplementary Tables 3-6).

According to the shared variants across the seven affected individuals, it is notable that loci in more than forty genes were indicated to be altered compared to those in a reference panel after restriction to a frequency <0.01, although variants in some genes may be artifacts by genome re-sequencing with short NGS reads (Supplementary Table 8). An INDEL in the 5′-UTR region of the *RTCA* gene and a SNP in the 3′-UTR region of the *ONECUT2* gene were suggested to be shared in seven individuals, although no previous reports regarding these genes and psychoses were found; thus, specific correlations between psychotic disorders remain undetermined. Notably, one gene shared by families 2 and 3 is the *GON4L* gene, which localizes in the cell nucleus. This gene is essential for hematopoiesis in animal models and is co-expressed with HDAC genes^29^.

Another hypothesis regarding HLA yielded an interesting result: 08:02, 09:01, and 15:01 in HLA-DRB1 were strongly associated with SLE in the Japanese population, and the common frequencies among the Japanese were 4.2, 14.3, and 7.7%, respectively^30^. Based on this ratio, five out of seven individuals is a high prevalence. It is possible that autoimmune encephalitis occurs in patients with acute psychotic episodes^31^, but routine autoantibody testing (e.g., anti-dsDNA, antinuclear antibody, or NMDAR-Ab) does not occur, especially in the psychiatry department if the patients do not show typical symptoms, such as high fever, skin rashes, or seizures.

Two pathways are suggested to be involved in peripheral immune signaling and neuropsychiatric symptoms: neural and humoral pathways. In the brain tissue, activated microglia interact with proinflammatory cytokines (IL-6, TNF-alpha, or IL-1beta), increasing oxidative stress and cortisol levels. This activation leads to neurodegeneration and psychotic symptoms in the brain. In the ventricle, activated macrophages with Toll-like receptors release proinflammatory cytokines. Both pathways should be verified at the genetic and proteomic levels in the future, although our current attempt revealed a new relationship between certain psychotic features and immunological vulnerability at the genetic level.

SZ appears to have its genetic background, at least in part, in the MHC region, according to several GWAS^19,32^. Focusing on a specific clinical phenotype will be key to elucidating the genetics underlying complex psychotic traits.

## Electronic supplementary material

Supple_table_1

Supple_table_2

Supple_table_3

Supple_table_4

Supple_table_5

Supple_table_6

Supple_table_7

Supple_table_8

Supple_table_9
